# Can SARS-CoV-2 Induce Uterine Vascular Anomalies and Poor Contractile Response?—A Case Report

**DOI:** 10.3390/medicina57070670

**Published:** 2021-06-29

**Authors:** Anca Lesnic, Bashar Haj Hamoud, Mircea-Octavian Poenaru, Valentin-Tiberiu Moldovan, Radu Chicea, Romina-Marina Sima, Mihai Popescu, Liana Ples

**Affiliations:** 1 Department of Obstetrics and Gynecology, Carol Davila University of Medicine and Pharmacy, 050474 Bucharest, Romania; lesnic.anca@gmail.com (A.L.); mpoenaru69@gmail.com (M.-O.P.); romina.sima@yahoo.es (R.-M.S.); plesliana@gmail.com (L.P.); 2 “Bucur” Maternity, Saint John Hospital, 012361 Bucharest, Romania; 3 Department of PhD Studies, IOSUD, Carol Davila University of Medicine and Pharmacy, 020021 Bucharest, Romania; 4 Department for Gynaecology, Obstetrics and Reproductive Medicine, Saarland University Hospital, Kirrberger Straße 100, Building 9, 66421 Homburg, Germany; bashar.hajhamoud@uks.eu; 5 Department of Pathology “Victor Babeş”, National Institute for Research and Development in Pathology and Biomedical Sciences, 050096 Bucharest, Romania; vali.valentintiberium@gmail.com; 6 Medicine Faculty, Lucian Blaga University, 550024 Sibiu, Romania; radu.chicea@gmail.com; 7 Department of Anaesthesia and Critical Care, Carol Davila University of Medicine and Pharmacy, 020021 Bucharest, Romania

**Keywords:** gravida, pregnancy, hypertension, COVID-19, SARS-CoV-2, atony of uterus, postpartum hysterectomy

## Abstract

We are reporting a case of a 36 year-old Severe Acute Respiratory Syndrome Coronavirus-2 (SARS-CoV-2) positive hypertensive primigravida with postpartum uterine atony that required emergency subtotal hysterectomy at Saint John Hospital Bucur Maternity Bucharest. The maternity was designated as the Coronavirus Disease 2019 (COVID-19) Maternity for Bucharest and Ilfov County since March 2020. The patient was mildly symptomatic for SARS-CoV-2, infection confirmed with reverse transcription polymerase chain reaction (RT-PCR). The caesarean section was performed and a live male fetus was born, 2630 g and Apgar Score of 9 (the male fetus was negative for SARS-CoV-2). Postpartum hysterectomy with adnexal preservation was performed because of uterine atony. The postoperative evolution was favorable. The patient was discharged with her baby 10 days after birth. Given the limited resources, the placenta, the umbilical cord and the uterus were not tested for SARS-CoV-2. The pathology exam revealed that on the maternal side there were specific uterine atony lesions as well as endometrial and miometrial ischaemia. The placenta had nonspecific findings: chronic ischemic lesions with small villi, fibrin deposits in the materno-fetal interface. The peculiarity of the case is that we report the morphological findings of the placenta and uterus resulted from intrapartum uterine atonia in a patient with gestational arterial hypertension, premature birth and COVID-19. Further studies are required to characterize the pattern of such intricate conditions.

## 1. Introduction

Coronavirus disease (COVID-19) is caused by a novel coronavirus, severe acute respiratory syndrome coronavirus 2 (SARS-CoV-2). Its outbreak was declared as a pandemic by the World Health Organization (WHO) in March 2020 [1]. As of 7 September 2020, approximately 27 million people were confirmed to have been infected and tested positive for SARS-CoV-2, with over 880,994 deaths worldwide reported to the WHO [2,3]. Allegedly, the virus was first identified in the respiratory tract of patients with pneumonia in Wuhan, Hubei province, China in December 2019, which was then indicated as a newly identified β-coronavirus. However, reports have suggested an earlier circulation of the virus [4,5]. SARS-CoV-2 diameter is approximately 65–125 nm, contains single strands of RNA, and has crown-like spikes on its outer surface. [6]. Patients may be asymptomatic or develop mild or severe COVID-19 symptoms [7].

The impact of the virus in pregnant women is of significant interest. In general, pregnant women are usually tested for SARS-CoV-2 infection, on hospital admission for delivery; most of them are often asymptomatic. Typically, the clinical presentations and severity of COVID-19 during pregnancy are similar to the disease spectrum observed in non-pregnant adults; however, some critical cases have been reported [7,8].

## 2. Case Presentation

We have presented a case of a 36-year-old woman from Bucharest who was admitted in our clinic for fever and chills that had started three days earlier. The case report was written following The CARE Guidelines: Consensus-based Clinical Case Reporting (CARE) and the patient signed the informed consent for the publication of the data [9]. She was 36 weeks pregnant. Polymerase chain reaction (PCR) test results on 4 May 2020 confirmed that the patient was positive for SARS-CoV-2. Hence, she was admitted to the hospital on the same day. The patient was treated with ceftriaxone (1 g/12 h intravenous) and azithromycin (500 mg orally/day). Five days later (9 May 2020), the patient was transferred to our maternity for uterine contractions. The patient had a significant medical history—one cone biopsy of the cervix in 2017 (no additional medical documents were presented by the patient), a right breast biopsy in 2010 (confirmed benign lesion), and gestational hypertension diagnosed at 30 weeks of pregnancy. Chronic medications included progesterone 600 mg per day vaginally, No Spa 40 mg twice a day orally, and nifedipine cp 20 mg twice a day orally. The pregnancy was supervised by a qualified specialist obstetrician. The gravida underwent all the required blood work and ultrasound examinations during the pregnancy. The combined first trimester test results revealed a low risk of chromosomal anomalies.

On admission, the patient was in a state of general good health, without respiratory symptoms or fever. Uterine contractions occurred at an interval of 2–3 min with minimal cervical changes (the cervix had multiple scarring from the cone biopsy). Ultrasound examination findings revealed oligohydramnios, and the blood test results revealed unremarkable findings. Considering the cervical dystocia and associated morbidities, cesarean section was performed. The newborn male weighed 2630 g and had an Apgar score of 9. The baby tested negative for SARS-CoV-2 at birth and re-tested 8 days after, in line with our testing protocols. After placenta delivery, uterine contraction was absent even after administration of 10 International Units (IU) oxytocin and ergometrine. Heavy bleeding and lack of efficient contractility (uterine atony) required an emergency subtotal hysterectomy with adnexal preservation. The immediate postoperative evolution was satisfactory with antibiotic and anticoagulant treatment, and the patient received 2 units of blood. Pulmonary Rx performed on the third day postoperatively revealed a left laterobasal condensation process and the decision to add hydroxychloroquine (200 mg/day orally) was made by an infectiologist (in line with the national protocol). The patient was discharged with her baby 10 days after birth (with two negative RT-PCR SARS-CoV-2 tests) and made a full recovery.

The uterus, placenta, and umbilical cord were evaluated at the pathology department. On gross examination, the uterus specimen measured 40 cm × 30 cm × 20 cm and weighed 1355 g, with marked areas of congestion and overall low consistency. The placenta had a diameter of 23 cm, and the umbilical cord measured 50 cm. A diffuse deposit of fibrinoid and small adherent blood clots was noted. The specimens were fixed using formaldehyde (4%) according to routine procedures—tissue processing using an automatic Leica Peloris3 station and paraffin inclusion. The resulting slides were hematoxylin and eosin stained on an automatic line (Leica ST5010-CV5030, Leica Biosystems^®^, Buffalo Grove, IL, USA). Additionally, immunohistochemistry was performed to evaluate the hyperplastic trophoblastic and microvascular processes described in the literature using CD-34 QBEnd/10 and AE1–AE3 polyclonal antibodies on an immunostainer (Leica Bond3, Leica Biosystems^®^, Buffalo Grove, IL, USA). The corresponding antigens and the counterstain were subsequently stained using Harris hematoxylin. The slides were examined using an Olympus BX 45 microscope with an ICC50W camera (Olympus^®^, Tokyo, Japan), and the slides were scanned using an AperioAT2 turbo station (Leica Biosystems^®^, Buffalo Grove, IL, USA).

**Figure 1:**
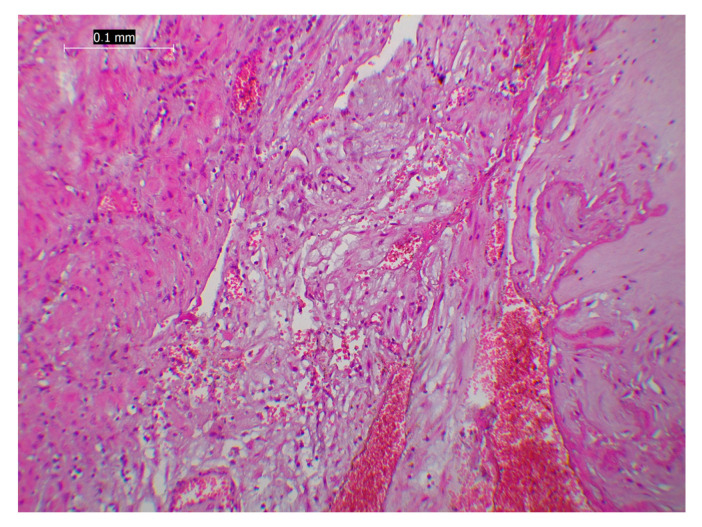
Miometrial-decidual interface, areas of blood suffusion, stromal necrosisa as well as fibroid depositions—Standard Hematoxylin Eosin (HE) Coloration 4×.

The histopathological examination of the uterus revealed parceled necrosis with luminal dilation of the arterioles in the endometrium (Figure 1), generalized edema of the myometrium, and significant congestion of the middle and small caliber vessels.

**Figure 2:**
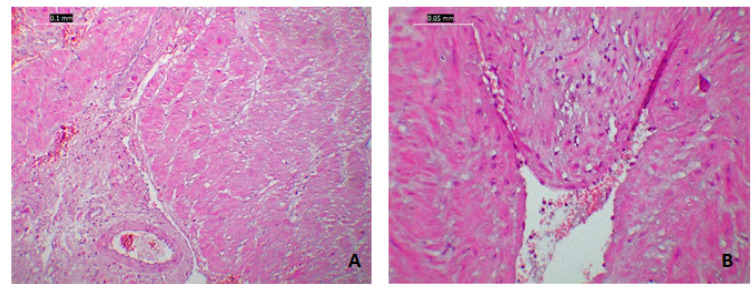

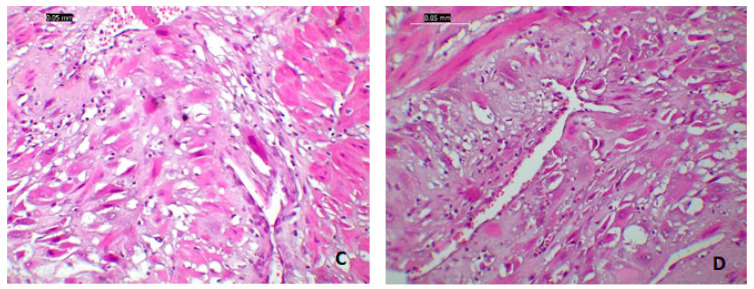
Histological modifications of the miometrium in a patient with SARS-CoV-2: (**A**) microphotography (4×) of the miometrium with diffuse oedema predominantly located in the perivascular areas; (**B**–**D**) interstitial blood suffusion as well as marked difference in celular coloration due to cellular oedema.

The interstitial edema had dissociated the muscular fibers and the conjunctive matrix, with minimal mononuclear cell infiltrates and areas of blood suffusion. Similar findings were observed at the level of the myometrial vessels; generalized edema had a tendency of endothelial separation (Figure 1 and Figure 2).

At the level of the macroscopic lesions (pale and with low consistency), the microscopic findings were variable intumescent islands of smooth muscle cells with pale eosinophilic cytoplasm and pyknotic nuclei-necrotic aspects (Figure 2).

**Figure 3:**
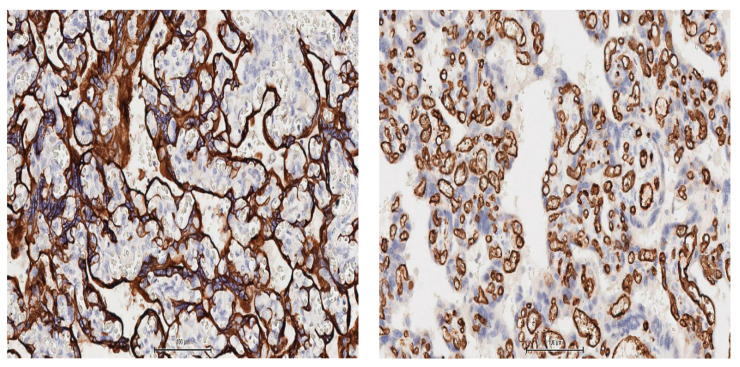
Sequential sections of the villi in paracentral area of the placenta, shows increased trophoblastic lining with eccentric hyperplasia as shown by the marquage with antibodies against pan- cytokeratins (clone AE1/AE3 cocktail, left panel) and marked vascular congestion (monoclonal antibodies against CD34; right panel). Counterstain Haematoxylin, 15X, image capture from a whole slide scan with AperioAT2Turbo.

The villous stroma appeared fibrotic with a marked vascular congestive appearance, as confirmed by immunohistochemistry (Figure 3).

**Figure 4:**
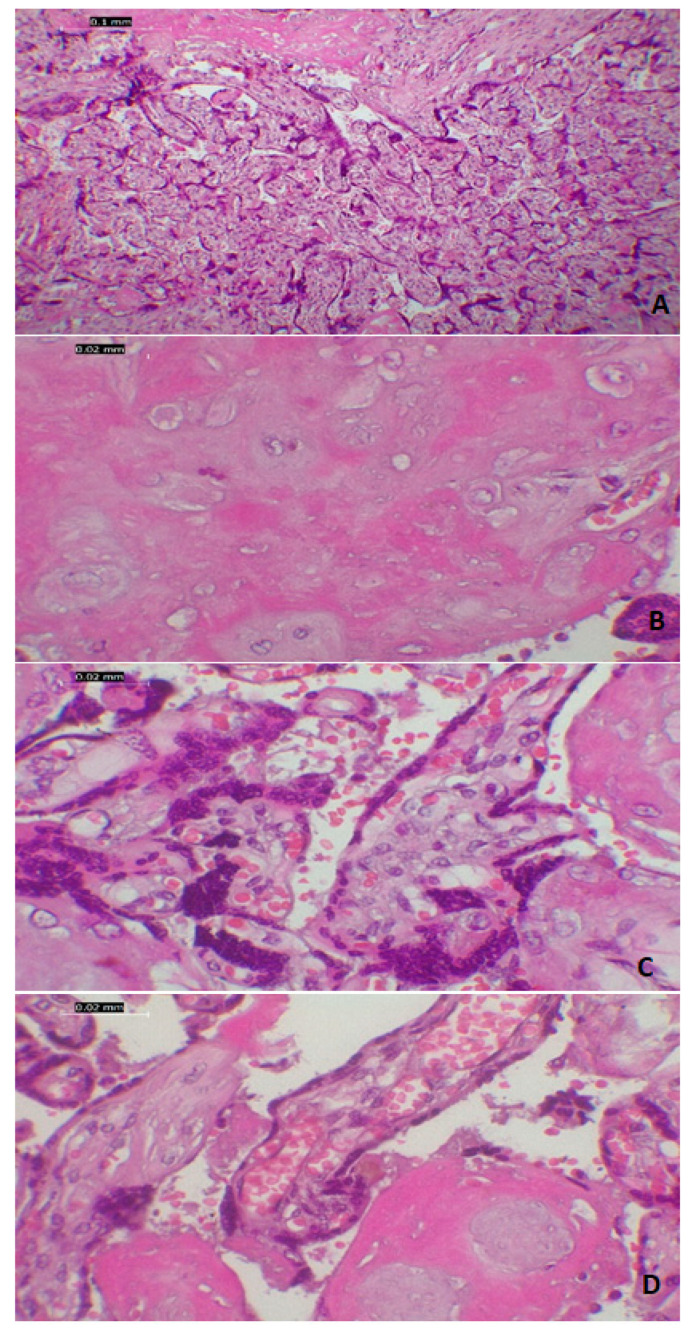
Microscopic aspect of placental modifcations seen in a positive SARS-CoV-2 patient: (**A**–**D**) villi deposits with paracentral small villi, densely packed with fibroid deposits that encompass the avascular villi, excentric trophoblastic hyperplasia with capilar hyperplasia and stasis at this level-all plead for chronic maternal ischaemia; Coloration HE, 20×.

The placental samples showed abundant deposits of fibrin located in the intervillous space, with areas of avascular villi and occasional hemosiderin deposits and calcifications. The villi appeared to be compressed with a small caliber and hyper-vascularity aspects. Notably, we found diffuse eccentrically trophoblastic hyperplasia, with trophoblastic detachments, without macrophagic multinucleated giant cells, neither significant atypia or viral nuclear inclusions (Figure 4).

In summary, the analysis of the specimens revealed the following facts:

- The mother had specific uterine atony lesions as well as endometrial and myometrial ischemia.
- Placental samples had nonspecific findings such as chronic ischemic lesions with small villi and fibrin deposits at the materno–fetal interface and trophoblastic hypertrophy/hyperplasia and vascular hyperplasia Light-microscopic procedures were shortened.

## 3. Discussion

Perinatal transmission of COVID-19 has been reported and discussed widely; however, the precise transmission route (transplacental, transuterine, or postpartum) is unclear [10]. In some cases, the placental samples test positive for SARS-CoV-2 on RT-PCR tests, but the neonates test negative [11,12]. In one particular case from Iran, the RT-PCR tests results were positive for the amniotic fluid sample and nasopharyngeal swab from the neonate (taken 24 h after birth) and were negative for the vaginal secretion sample, umbilical cord blood sample, and nasopharyngeal swab from the neonate (taken immediately after birth) [13]. Another case report described a possible transplacental transmission in a neonate with neurological symptoms born to a mother infected with SARS-CoV-2 in her last trimester of pregnancy [14].

Asymptomatic infections have also been reported in infants and neonates. In a review of 160 infants with confirmed COVID-19 [15], 16% infants were asymptomatic. There are limited data regarding the role of breastfeeding in transmission of COVID-19; hence, more studies are needed [16]. A systematic review and meta-analysis reported that preterm birth rates were higher in pregnant women with COVID-19 than in pregnant women in general [17], which is consistent with our experience at Bucur Maternity over the past 6 months. Histopathological data on the placenta, uterus, and umbilical cord are scarce, possibly because histopathological analysis requires costly facilities and extended studies.

Several studies have linked premature births with SARS-CoV-2 infection even in mild cases; however, the possible mechanisms are currently under discussion. Similarly, in our case, premature delivery was performed at 36 weeks of gestation due to SARS-CoV-2 infection, and the involvement of the virus in premature contraction induction can be considered [18,19,20].

The histopathological findings of the placenta, including chronic ischemic lesions with small villi, fibrin deposits in the materno–fetal interface, trophoblastic hypertrophy and hyperplasia, and vascular hyperplasia could be considered adaptive mechanisms in an environment of chronic ischemia. However, these changes have also been described in other viral infections of the placenta, such as Zika virus infection [21].

Uterine atonia can be theorized to be caused by a combination of chronic maternal hypertension, chronic treatment with nifedipine, SARS-CoV-2 infection, and preterm birth. The significant interstitial edema that dissociated the muscular fibers and the conjunctive matrix can be considered the most important factor leading to uterine contraction insufficiency. Recent studies have proposed the theory of postpartum acute myometritis, which is characterized by specific acute inflammatory changes in the uterine myometrium, such as infiltration of neutrophils and macrophages, increased number of complement C5a receptors in the interstitial area, anaphylactoid reaction, and massive stromal edema; however, the exact cause has not been discovered [22].

This case report highlights that subtle non-specific lesions of the maternal vascular endothelium have important clinical relevance and outcomes. Previous studies have reported these lesions are pseudo-atheromatous, with an intraparietal accumulation of macrophages and fibrinoid necrosis [23]. Although whether COVID-19 did cause those morphological findings and whether this changes and COVID-19-pathophysiology might be correlated cannot be identified; however, the data can be of used for future studiesconsidering that, so far, the placental morphological impact of COVID-19 is still unclear.

Given the novelty of the virus, evidence regarding the vertical transmission of the virus is scarce and additional studies are needed because of the important implications and severe complications [24].

The present study supports the conclusion of a recent meta-analyses where the authors proved that COVID-19 with at least one co-morbidity increases risk of intensive care and mortality [25]. However, new insights are showing the promising role of different therapies such as modulators of endocannabinoids receptor and alpha-lipoic acid in the management of COVID-19 related inflammation [26,27]. Further studies are required to prove the hypotheses and support the results with clinical, pathological and laboratory evidences.

## 4. Conclusions

COVID-19 is a systemic disease with various manifestations; the clinical manifestations of this disease in pregnant women, who are routinely tested for SARS-CoV-2 infection on hospital admission for delivery, are of great interest. Although these patients are often asymptomatic, it seems that the virus might have important consequences regarding the way of birth, postpartum recovery, and even fetal outcomes.

In conclusion, the manifestations observed in this case were similar to those reported in other cases of maternal SARS-CoV-2 infections. The peculiarity of the case is that we report the morphological findings of the placenta and uterus resulted from intrapartum uterine atonia in a patient with gestational arterial hypertension, premature birth and COVID-19. Further studies are required to characterize the pattern of such intricate conditions.

## Data Availability

All data are available on request to the corresponding author.
